# Volume-Assisted Estimation of Remnant Liver Function Based on Gd-EOB-DTPA Enhanced MR Relaxometry: A Prospective Observational Trial

**DOI:** 10.3390/diagnostics13183014

**Published:** 2023-09-21

**Authors:** Niklas Verloh, Carolina Rio Bartulos, Kirsten Utpatel, Frank Brennfleck, Andrea Goetz, Andreas Schicho, Claudia Fellner, Dominik Nickel, Florian Zeman, Johannes F. Steinmann, Wibke Uller, Christian Stroszczynski, Hans-Jürgen Schlitt, Phillip Wiggermann, Michael Haimerl

**Affiliations:** 1 Department of Radiology, University Hospital Regensburg, 93053 Regensburg, Germanymichael.haimerl@klinik.uni-regensburg.de (M.H.); 2 Department of Diagnostic and Interventional Radiology, Medical Center University of Freiburg, Faculty of Medicine, University of Freiburg, 79106 Freiburg, Germany; 3 Institut für Röntgendiagnostik und Nuklearmedizin, Städtisches Klinikum Braunschweig gGmbH, 38114 Braunschweig, Germany; 4 Department of Pathology, University Regensburg, 95053 Regensburg, Germany; 5 Department of Surgery, University Hospital Regensburg, 93053 Regensburg, Germany; 6 MR Applications Predevelopment, Siemens Healthcare GmbH, 91052 Erlangen, Germany; 7 Center for Clinical Trials, University Hospital Regensburg, 93053 Regensburg, Germany; 8 Department of Anesthesiology, University Medical Center Regensburg, 93053 Regensburg, Germany

**Keywords:** liver resection, liver dysfunction, postoperative remnant liver function, small for size syndrome, magnetic resonance imaging (MRI), gadoxetic acid-enhanced MRI (Gd-EOB-DTPA)

## Abstract

In the context of liver surgery, predicting postoperative liver dysfunction is essential. This study explored the potential of preoperative liver function assessment by MRI for predicting postoperative liver dysfunction and compared these results with the established indocyanine green (ICG) clearance test. This prospective study included patients undergoing liver resection with preoperative MRI planning. Liver function was quantified using T1 relaxometry and correlated with established liver function scores. The analysis revealed an improved model for predicting postoperative liver dysfunction, exhibiting an accuracy (ACC) of 0.79, surpassing the 0.70 of the preoperative ICG test, alongside a higher area under the curve (0.75). Notably, the proposed model also successfully predicted all cases of liver failure and showed potential in predicting liver synthesis dysfunction (ACC 0.78). This model showed promise in patient survival rates with a Hazard ratio of 0.87, underscoring its potential as a valuable tool for preoperative evaluation. The findings imply that MRI-based assessment of liver function can provide significant benefits in the early identification and management of patients at risk for postoperative liver dysfunction.

## 1. Introduction

While a potentially life-saving procedure, liver resection can occasionally lead to liver failure due to inadequate remaining functional organ capacity, known as “small for size syndrome” [1,2]. This major postoperative complication significantly contributes to morbidity, mortality, and healthcare costs, with liver failure increasing the expenses associated with liver resection by approximately threefold [3]. Such outcomes necessitate a precise preoperative evaluation of liver function to mitigate the risk of postoperative liver failure [4,5,6,7,8,9,10].

Current evaluations of liver function rely on liver volumetry and clinical and biochemical parameters assessment. However, these techniques, including biochemical parameters, clinical scoring systems, and dynamic quantitative liver function tests, have proven inadequate in predicting postoperative liver failure [11]. A meta-analysis revealed a substantial 14.1% incidence of postoperative liver failure, accompanied by a 2.1% mortality rate posthepatic resection at 90 days [12,13]. 

A common limitation of tests currently used is their assumption of uniform liver function distribution throughout the liver parenchyma, thereby suggesting liver functional reserve is solely volume-dependent. Emerging imaging techniques aiming to evaluate both whole and regional liver function can potentially enhance preoperative liver function assessment [14]. This was demonstrated by Haimerl et al., who successfully estimated total liver function (as quantified by the ICG-PDR) using Gd-EOB-DTPA-enhanced volume-assisted MR relaxometry [14]: Results from 107 patients indicated that there was a significant correlation between plasma disappearance rate of ICG (ICG-PDR) and MR relaxometry metrics, with the volume-assisted index showing a strong correlation (r = 0.92; *p* < 0.001). Haimerl et al. suggested that Gd-EOB-DTPA-enhanced MR relaxometry could be a robust, non-invasive method for quantifying liver function and potentially monitoring liver disease progression [14].

This promising development sparked our research interest in exploring the potential of assessing remnant liver function after resection using preoperative Gd-EOB-DTPA-enhanced liver MRI. Therefore, the primary study objective was to determine to what extent segmental liver function imaging with Gd-EOB-DPTA is possible and to compare it to the postoperative liver function after liver resection.

This study included patients scheduled for liver resection who underwent a liver MRI enhanced with Gd-EOB-DTPA as part of their preoperative preparation. These patients were further subjected to a liver function test both preoperatively (at the time of MRI) and postoperatively (within 12 h), using the indocyanine green clearance test as the standard of reference. Following the procedure, we investigated postoperative liver function using a combination of clinical and laboratory parameters as secondary objectives.

## 2. Materials and Methods

### 2.1. Study Design

This study was approved by the University Hospital Regensburg’s local institutional review board, ensuring that all the regulations and guidelines were followed. Written consent was obtained from the study participants.

One hundred and two patients were enlisted for participation. Of the initial pool, three patients were precluded from undergoing surgery due to either the advanced progression of their tumor or the discovery that their liver lesion was benign. In four instances, the full MRI protocol was not completed. During intraoperative contrast-enhanced ultrasound (CEUS), advanced tumor growth was detected in 12 patients, resulting in a therapy change: to either forgo the operation (*n* = 3) or proceed with only a sampling (*n* = 9). Intraoperative hypertrophy induction was performed for one patient to reduce the risk of postoperative liver failure.

The patient cohort, examined from October 2016 to January 2020, required liver resection for a variety of reasons: the presence of one or more liver metastases (*n* = 32 cases), hepatocellular carcinoma (*n* = 21 cases), cholangiocellular carcinoma (*n* = 15 cases), benign liver lesions (*n* = 5 cases), inflammatory/parasitic issues such as echinococcosis (*n* = 2 cases), lymphoma (*n* = 1 case), and various other conditions such as polycystic liver lesions (*n* = 6 cases).

**Figure 1:**
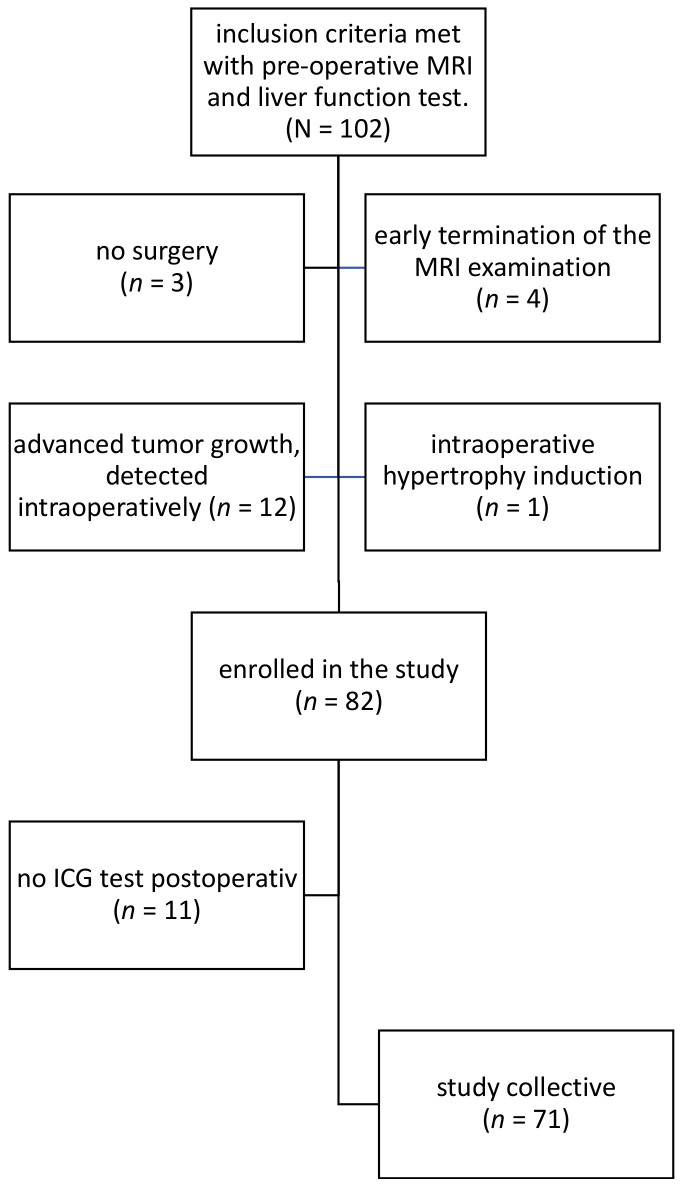
Flowchart depicting patient inclusion in the study cohort. This diagram elucidates the inclusion and exclusion process for determining the final study cohort. The chart visually illustrates the step-by-step selection procedure and the various exclusion criteria leading to the final study population (*n* = 71). The exclusion criteria include advanced tumor progression prohibiting surgery, benign liver lesions negating the need for surgery, inability to complete the full MRI protocol, intraoperative findings necessitating change in surgical plan, and the failure to perform the Indocyanine Green (ICG) test both pre- and postoperatively due to various factors such as clinical instability and patient mortality.

Another limiting factor in this study was the inability to perform the Indocyanine Green (ICG) test within the 12 h post-surgery window for 11 patients. These constraints stemmed from various causes, such as clinical instability (*n* = 2), inadequate capillary blood supply to peripheral extremities leading to erroneous ICG test results (*n* = 8), or the unfortunate immediate death of one patient. Consequently, the primary study cohort for analyzing changes in liver function consisted of the remaining 71 patients, for whom both pre- and postoperative ICG findings were available. The inclusion and exclusion criteria are shown in Figure 1.

This study adopted a simplified criterion to identify instances of postoperative acute liver dysfunction [2]: an increase in either serum bilirubin (cut-off < 1.0) or INR (cut-off > 1.15) on days 3 to 7 postoperative. A clinical indication of severe liver failure was considered if the 50/50 criterion was met (serum bilirubin > 50 mmol/L = 2.92 mg/dL and INR > 1.7) or if there was a postoperative peak bilirubin level exceeding 7 mg/dL within the same period.

**Figure 2:**
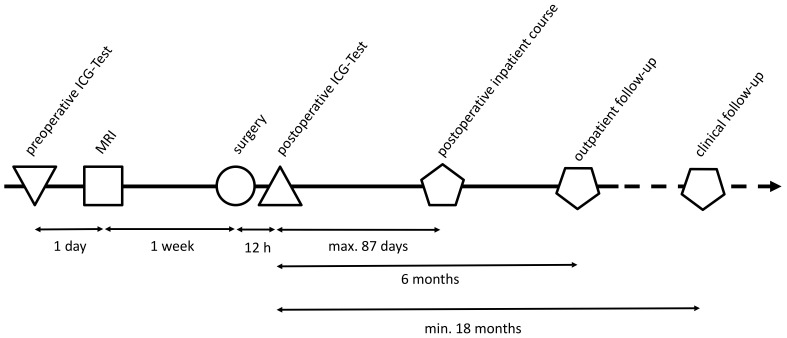
Timeline of the study cohort. This figure presents a graphical timeline of the cohort’s key study activities and intervals. The preoperative Indocyanine Green (ICG) test was conducted one day before the MRI scan. Following this, the MRI scan and the liver operation are performed with a one-week interval in between. Within 12 h postoperatively, a second ICG test is administered, and the maximum inpatient postoperative period spanned up to 87 days. Upon discharge, patients were scheduled for outpatient follow-up for a period of six months after the operation. The clinical follow-up was conducted at least 18 months after the operation. This timeline provides a succinct overview of this study’s procedural chronology.

As part of the secondary study objectives, patients were monitored for a minimum of six months to screen for potential subacute liver failure (28 days to 6 months postoperative) or chronic liver dysfunction (>6 months). The mortality rate was also tracked until July 2021 (Figure 2).

### 2.2. Indocyanine Green (ICG) Test Procedure and Classification

The ICG Plasma Disappearance Rate (PDR) was evaluated using the non-invasive LiMON pulse-densitometric system (Impulse Medical System, Munich, Germany). A 0.5 mg/kg body weight bolus dose of ICG (ICGPulsion, Munich, Germany) was administered intravenously, immediately followed by a 10 mL saline flush. Monitoring was accomplished through an ICG finger clip linked to the liver function monitor (LiMON) by an optical probe. The ICG was detected based on fractional pulsatile variations in optical absorption. ICG-PDR values were computed via a monoexponential transformation of the original ICG concentration curve and its backward extrapolation to the initial point, thereby describing the decay rate as a percentage over time.

Patient classification prior to the operation followed Haegele et al.’s guidelines [15], where a preoperative PDR of less than 17%/min or R15 greater than 8% were established as the cut-off points for liver dysfunction (LDF) and normal liver function (NLF).

For postoperative assessments, patients were further divided into three categories depending on their ICG results:

- Postoperative Liver Dysfunction (PLDF) was defined for patients with a PDR of less than 10%/min;
- Patients with a PDR between 10 and 17%/min were considered at risk for PLDF;
- A postoperative PDR value greater than 17%/min indicated Postoperative Normal Liver Function (PNLF).

### 2.3. Image Acquisition and Processing

All MRI imaging procedures were executed using a clinical 3 Tesla whole-body system (MAGNETOM Skyra; Siemens Healthcare, Erlangen, Germany). Signal reception was facilitated by a combination of body and spine array coil elements (18-channel body matrix coil, 32-channel spine matrix coil). The liver-specific contrast agent Gd-EOB-DTPA (Primovist^®^; Bayer AG, Berlin, Germany) was administered via bolus injection (0.1 mL/kg body weight) at a flow rate of 1 mL/s, followed by a 20 mL NaCl flush.

T1 mapping was carried out both prior to and 20 min following contrast administration, employing a T1-weighted volume-interpolated breath-hold examination (VIBE) research sequence with a repetition time (TR) of 5.79 ms and two echo times (TE1 and TE2) of 2.46 ms and 3.69 ms, respectively [16]. The employed method was based on a three-dimensional spoiled-gradient echo sequence with variable flip angles (1°, 7°, and 14°), achieving a voxel size of 3.6 mm × 2.5 mm × 4.7 mm, which was interpolated to 1.3 mm × 1.3 mm × 3.0 mm.

To enhance the uniformity of the T1 maps, a B1 map of the liver was captured for each patient before the T1 relaxometry measurements were taken, enabling B1 correction [17]; inline computation was utilized to generate color-coded T1 maps. Leveraging the controlled aliasing in parallel imaging results in a higher acceleration (CAIPIRINHA) technique with an acceleration factor of 4; it was possible to cover the entire liver within a single breath-hold, with an acquisition time of 17 s.

Image processing was performed utilizing the open-source Horos imaging software (Horos Project, Annapolis, MD, USA). The liver volume (LV) was assessed based on corresponding MR images in the hepatobiliary phase through manual segmentation, excluding observable vessels and liver lesions. This process was supported by a semi-automated region-growing algorithm with manual edge correction, implemented through the open-source software OsiriX [18].

### 2.4. Model for Estimating Liver Function and Remaining Postoperative Liver Function

Gd-EOB-DTPA uptake was quantified by calculating the reduction rate in T1 relaxation time between the pre-contrast (T1plain) and the Gd-EOB-DTPA-enhanced phase (T1HBP). The formula used to determine the reduction rate in the T1 relaxation time (RR) was:

RR = ((T1plain − T1HBP)/T1plain) × 100%(1)

The liver volume (LV) and the reduction rate (RR) were then used to estimate liver function, as described by Haimerl et al. [14]. In this model, the liver function is estimated based on changes in T1 relaxation time and liver volume, providing a valuable tool to predict liver function.

Estimated Liver Function (eLF) = 0.84 × e^(0.038 × RR)^ × e^(0.045 × LV [mL])^ (2)

**Figure 3:**
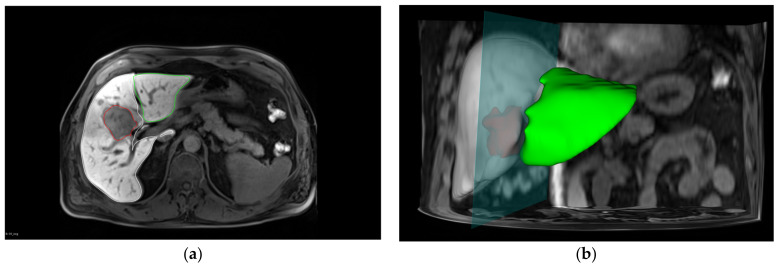
Virtual operation planning. (**a**) Axial MRI scan of the liver in the hepatobiliary phase using a VIBE sequence. The liver contour is outlined to facilitate liver segmentation. The tumor region is represented in red, the resected liver lobe in white, and the remaining liver lobe in green. (**b**) A 3D visualization of the virtual operation planning, providing a comprehensive spatial understanding of the liver structure pre- and post-operation, as well as the location and extent of the tumor and the resected liver lobe.

A collaborative effort with surgical colleagues facilitated the performance of segmentation according to the surgical report, allowing for the estimation of the remaining liver volume post-surgery (Figure 3). To ensure accuracy, the remaining liver volume was determined by subtracting the measured resected liver volume from the total liver volume, which can be represented as:

Remaining liver volume (rLV) = LV − resection volume(3)

To estimate the remaining postoperative liver function, an adaption of formula (2) was used, in which the LV was exchanged with rLV:

Estimated Liver Function (erLF) = 0.84 × e^(0.038 × RR)^ × e^(0.045 × rLV [mL])^ (4)

Postoperative liver dysfunction was determined and classified based on the estimated remaining liver function (erLF) derived from the MRI analysis. This classification followed the methodology employed in the ICG test evaluation, providing a consistent framework for assessing liver function across different testing modalities. The precise criteria and cut-offs for normal function, at-risk function, and dysfunction were in line with the established standard.

### 2.5. Statistical Analysis

All statistical computations were performed using IBM SPSS Statistics software (version 29, Chicago, IL, USA). Data are presented as mean ± standard deviation (SD). In assessing the ability of each model to predict postoperative liver dysfunction compared to ICG results, we calculated sensitivity, specificity, positive predictive value (PPV), and negative predictive value (NPV). The non-parametric Mann–Whitney U test was employed to analyze differences between the groups. Survival analysis was carried out using a univariable Cox proportional hazards model to evaluate the predictive value of the estimated postoperative liver function on overall survival. The Hazard ratio (HR) and the 95% confidence interval (CI) are provided as effect estimates. We employed a Kaplan–Meier plot to represent the survival analysis visually. 

All tests performed were two-tailed, and a *p*-value of less than 0.05 indicated statistical significance.

## 3. Results

This investigation involved the analysis of 71 liver resections. The procedures included 21 atypical liver resections, 14 segment resections, and 36 hemihepatectomies (5 left, nine extended-left, 12 right, and 10 extended-right). The mean volume of resected liver tissue was 508.1 ± 512.5 mL, and the average operation time was approximately 4.2 ± 1.9 h. No significant difference was observed between the volume of liver tissue virtually planned (501.6 ± 507.9 mL) for resection, and the actual volume resected during the procedure. 

In the analyzed cohort of 71 patients who underwent liver resection, the mean age was 62.8 years, with a standard deviation of 14.5 years. With regard to body composition, the average Body Mass Index (BMI) stood at 25.4 with a standard deviation of 4.2, suggesting a diverse mix of body types within the patient population. Patient characteristics are presented in Table 1.

Examining preoperative liver function, as determined by the ICG test, the mean PDR was 20.2%/min, and the mean Retention Rate at 15 min (R15) was 7.7%. The analyzed cohort displayed a range of pre-existing liver conditions. Twenty-two patients demonstrated pre-existing LDF, as revealed by the ICG test conducted at the time of the MRI before surgery. The RR in the T1 relaxation time significantly varied between patients with pre-existing LDF and those with NLF, with LDF patients showing a lower RR value (51.9 ± 11.8%) compared to NLF patients (61.3 ± 8.6%, *p* = 0.002). No significant difference was observed in liver volume between these two groups (*p* = 0.567). However, a significant difference was apparent in the eLF between the LDF group and the NLF group (*p* ≤ 0.001).

Postoperatively, the average PDR fell slightly to 17.5%/min, and the R15 increased to 14.5%. Postoperative ICG test results provided further stratification of the patients into three groups: those with normal liver function postoperatively (PDR > 17%/min, *n* = 32), those with postoperative liver dysfunction (PDR < 10%/min, *n* = 19), and those at risk for postoperative liver dysfunction (PDR 10–17%/min, *n* = 20). Intriguingly, these postoperative ICG results significantly correlated with the preoperative ICG values (*p* ≤ 0.001).

Further, a comparison of the various parameters, such as T1 plain, T1 HBP, and liver volume among these three postoperative groups, did not yield significant differences. However, a significant difference was observed in T1 RR, erLF, and rLV. Despite the variations observed in the different patient groups, posthoc analysis revealed no significant differences between postoperative PDR values in the range of 10–17 %/min and those below 10%/min for parameters like pre- and operative PDR and R15, T1 RR, eLF, RLV, and erLF.

### 3.1. Predicting Risk for or Postoperative Liver Dysfunction

This study sought to develop a model to predict the risk of postoperative liver dysfunction by estimating the remaining liver function after surgery; this was then compared to an existing predictive model that relied on preoperative ICG test results [15]. For these comparisons, a postoperative PDR value of less than 10%/min was set as the cut-off.

When differentiating between PLDF and PNLF, the existing model based on the preoperative ICG test yielded an AUC value of 0.73, with an overall accuracy of 0.70. This model exhibited a sensitivity of 0.53, a specificity of 0.77, a positive predictive value of 0.46, and a negative predictive value of 0.82.

On the other hand, the newly proposed model for estimating remaining postoperative liver function demonstrated improved predictive performance. It delivered an accuracy of 0.79, a sensitivity of 0.53, a specificity of 0.89, a positive predictive value of 0.63, and a negative predictive value of 0.84. The AUC for this model was 0.75, with no statistical difference to the model based on preoperative ICG (Table 2). 

### 3.2. Clinical Follow-Up

During the clinical follow-up, six out of 32 patients with a postoperative ICG value (PDR) greater than 17%/min showed signs of acute liver dysfunction. Notably, no acute or subacute liver failure cases were observed in this group. Five patients with a postoperative ICG PDR value between 10 and 17%/min (*n* = 20) displayed signs of acute liver dysfunction, which recovered in the following six months.

In the group of patients with a PDR less than 10%/min postoperatively (*n* = 19), all three cases of liver failure were correctly identified by the postoperative ICG test. In this group, 12 patients exhibited acute liver dysfunction, while four showed no clinical signs of postoperative liver dysfunction. Of the patients with acute liver dysfunction, seven patients recovered entirely in the following six months, while the others showed evidence of continued decreased liver function.

The newly proposed model proved its efficacy by correctly predicting all instances of liver failure. Furthermore, it successfully predicted liver synthesis dysfunction in 10 out of 23 cases; this corresponded to an overall accuracy of 0.78, with a sensitivity of 0.50, a specificity of 0.93, a positive predictive value of 0.81, and a negative predictive value of 0.76. Thus, the proposed model demonstrated high reliability in predicting both liver failure and liver synthesis dysfunction.

### 3.3. Survival Analysis

**Figure 4:**
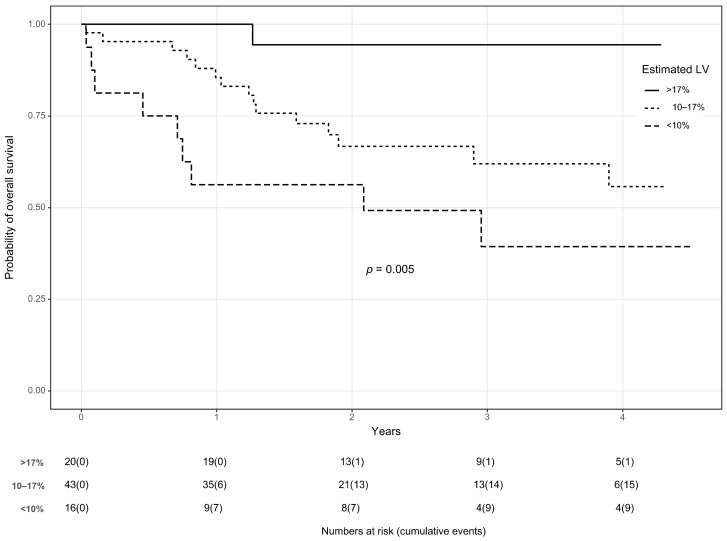
Kaplan–Meier Survival Analysis. This plot demonstrates the survival probabilities based on the estimated remaining liver function (erLF) post-resection. The solid line represents patients with erLF greater than 17, the small-dotted line represents patients with erLF between 10 and 17, and the dashed line represents patients with erLF less than 10. The differentiation in survival probabilities among these groups illustrates the predictive value of erLF on patient survival post-liver resection.

The survival analysis conducted in this study demonstrated the utility of the proposed model, which includes the estimated remaining liver function (erLF) as a significant predictor for overall survival (Figure 4). The model yielded a hazard ratio (HR) of 0.87 (95% Confidence Interval: 0.80; 0.95), suggesting a strong statistical significance with a *p*-value of 0.003.

This model also clearly differentiated overall survival when categorizing erLF into three groups: greater than 17, between 10 and 17, and less than 10.

## 4. Discussion

MRI, with the hepatobiliary-specific contrast agent Gd-EOB-DTPA, has been established as a valuable imaging modality for the liver. It offers precise anatomical information, along with clear visualization of liver lesions. As such, it has become a standard tool in preoperative assessment [19,20,21]. In addition to this role, it has also shown potential in experimental liver function assessment [14,22,23,24]. Our findings reveal that liver function assessment via MRI is consistent with preoperative ICG results. This consistency may be attributed to the hepatocyte-specific contrast agent Gd-EOB-DTPA undergoing similar metabolic pathways to ICG in vivo [25,26]. 

The results of our study align with previous research, confirming the efficacy of Gd-EOB-DTPA-enhanced MRI for imaging functional liver reserve [27,28,29,30]. Moreover, our data suggest that coupling this approach with liver volume analysis can yield even more accurate quantifications of liver function [14]. Notably, our study used T1 relaxometry to quantify Gd-EOB-DTPA uptake, unlike many of the cited studies. Both signal intensity (SI) measurements and T1 relaxometry can reliably assess liver function; however, the latter exhibit certain key advantages [20,31]. For instance, SI measurements are sensitive to technical parameters like receiver coils and radiofrequency amplifiers, impairing direct comparisons between individual patients or examinations [32]. T1 relaxometry, however, bypasses these issues. Its utility for liver function evaluation is further supported by its significant correlation with established liver function tests and scores, including the Child-Pugh score [32,33], the MELD score [23], and the ICG clearance test [14,34].

Interestingly, many of our participants with pre-existing liver dysfunction underwent liver resection, mainly because our center is a special center for liver surgery, often attracting more complex cases with compromised liver function. However, the expertise of our surgical department likely helped maintain a relatively low occurrence of acute liver failures. Furthermore, our study design mandated that physicians were alerted to any abnormal postoperative ICG test results, enabling close monitoring and the implementation of countermeasures where necessary.

Typical surgical planning incorporates total bilirubin level, Child-Pugh score, ICG R15, and remaining liver volume or volume ratio. Decision trees then combine these parameters to identify high-risk patients [35]. However, our study, among others, has demonstrated that liver function distribution among patients with liver disease can often be quite uneven [36,37]. Existing criteria fail to assess this inhomogeneity, thereby limiting their accuracy directly. Gd-EOB-DTPA-enhanced MRI, on the other hand, offers a means to assess regional liver function with high resolution. Thus, merging remnant liver volume with regional liver function parameters derived from dynamic Gd-EOB-DTPA-enhanced MRI may yield superior results than traditional parameters. Therefore, we solely relied on MRI to quantify remnant liver function and volume in this study. This approach circumvents the challenges in previous studies that relied on misregistered CT and MRI images to calculate remnant liver function [29,30,38,39,40]. Additionally, our study followed a standardized MRI and liver function evaluation protocol as a prospective trial. 

Postoperative liver function was assessed in patients based on the ICG test, and patients were categorized into three groups. Although no significant differences were found between these groups regarding baseline T1 plain and T1 HBP, our study revealed the potential value of T1 RR, eLF, and erLF measurements in liver function prediction.

Our findings align with a retrospective cohort study by Wang Y. et al., which utilized preoperative routine clinical dynamic Gd-EOB-DTPA-enhanced MRI to predict post-hepatectomy liver failure [37]. Similarly to their study, our prospective observational trial found the Gd-EOB-DTPA enhanced MR relaxometry to be a valuable tool in predicting postoperative liver function with an improvement over the ICG-based model. Our proposed model offered an accuracy of 0.79 with a sensitivity of 0.53, a specificity of 0.89, and a slightly higher AUC of 0.75, showing better overall predictive performance. Compared to Wang, Y et al., our study further emphasizes the potential utility of the volume-assisted estimation of the remnant liver function model in predicting overall survival and suggesting its potential long-term impact on patient outcomes.

Although our findings are promising, the limitations of this study should be recognized. Firstly, this study was conducted at a single center specializing in liver surgery, potentially biasing the results due to the center’s high level of surgical experience and the complexity of cases handled; this may limit the generalizability of our results to other settings. Second, despite our model’s predictive solid performance, it should be noted that the accuracy, sensitivity, and specificity values were not exceedingly high, highlighting room for further refinement and enhancement of this model. Moreover, while our model successfully predicted all instances of liver failure, it could only predict acute liver dysfunction, although limited and in most cases not clinically relevant, in less than half of the cases, suggesting potential limitations in its predictive power.

Furthermore, this study’s design must also be mentioned as an influencing factor: In the case of abnormal values in the postoperative ICG test, the attending physicians were informed accordingly, so close monitoring was often carried out, and countermeasures were initiated in the case of clinically relevant changes. Lastly, while we identified cut-off values appropriate for a Western population, these may not apply to other populations with different genetic and environmental backgrounds. As such, future studies with more diverse patient cohorts are needed to validate and further refine our proposed model.

## 5. Conclusions

Our results show that MRI liver function assessment was significantly linked to postoperative liver failure and mortality. As part of this study, we identified cut-off values (erLF > 17, 10–17, <10) appropriate for a Western population to accurately flag high-risk patients likely to develop postoperative acute liver dysfunction or liver failure.

The findings highlight the potential clinical implications of incorporating erLF into assessing and managing patients undergoing liver procedures. This approach has the potential to aid in the early identification and management of patients at risk for postoperative liver dysfunction, allowing for timely intervention and improved patient outcomes. In addition, by considering the estimated remnant liver function, clinicians can obtain valuable prognostic information regarding overall survival; this may enable more personalized treatment strategies and closer monitoring for patients at higher risk, ultimately improving (long-term) patient outcomes.

## Figures and Tables

**Table 1:** Patient characteristics of the study collective.

	Study Collective (*n* = 71)	Preoperative ICG Results			Postoperative ICG Results			
		NLF (*n* = 49)	LDF (*n* = 22)	*p*-Value	PDR >17%/min (*n* = 32)	PDR 10–17%/min (*n* = 20)	PDR <10%/min (*n* = 19)	*p*-Value
Age (years)	62.8 ± 14.5	60.1 ± 15.8	68.0 ± 9.8	0.079	57.8 ± 16.7	66.0 ± 12.2	67.1 ± 11.0	0.105
Weight (kg)	75.8 ± 15.6	73.7 ± 14.5	79.7 ± 17.2	0.184	75.5 ± 13.3	74.7 ± 17.0	77.5 ± 18.1	0.963
Height (meter)	1.72 ± 0.1	1.73 ± 0.1	1.71 ± 0.1	0.495	1.74 ± 0.1	1.72 ± 0.1	1.71 ± 0.1	0.834
BMI	25.4 ± 4.2	24.4 ± 3.7	27.1 ± 4.6	0.20	25.0 ± 3.2	25.2 ± 4.0	26.4 ± 5.8	0.726
PDR pre (%/min)	20.2 ± 6.8	23.4 ± 5.3	13.0 ± 3.4	≤0.001	23.4 ± 6.8	18.7 ± 4.1	16.2 ± 6.7	≤0.001
R15 pre (%)	7.7 ± 8.4	3.7 ± 1.9	16.4 ± 10.5	≤0.001	4.5 ± 4.6	7.1 ± 4.0	13.5 ± 13.0	≤0.001
PDR post (%/min)	17.5 ± 10.7	20.3 ± 11.4	11.4 ± 5.0	≤0.001	26.3 ± 9.7	13.8 ± 1.6	6.7 ± 2.0	≤0.001
R15 post (%)	14.5 ± 14.5	10.6 ± 12.9	22.9 ± 14.4	≤0.001	3.3 ± 2.3	13.0 ± 3.4	34.3 ± 12.2	≤0.001
T1 plain (ms)	751.9 ± 99.7	764.5 ± 93.1	723.9 ± 110.2	0.273	771.2 ± 93.9	730.8 ± 76.5	741.8 ± 126.6	0.363
T1 HBP (ms)	310.2 ± 81.0	294.4 ± 68.2	345.3 ± 96.7	0.033	286.7 ± 64.5	314.5 ± 58.6	345.2 ± 111.4	0.237
T1 RR (%)	58.4 ± 10.6	61.3 ± 8.6	51.9 ± 11.8	0.002	62.6 ± 8.9	56.8 ± 7.9	53.1 ± 13.0	0.010
LV (ml)	1609 ± 443	1641 ± 474	1537 ± 365	0.567	1662 ± 493	1595 ± 363	1536 ± 442	0.679
eLF	17.5 ± 7.5	19.4 ± 7.6	13.2 ± 5.3	≤0.001	20.6 ± 8.1	15.4 ± 4.2	14.5 ± 7.7	0.007
rLV (ml)	1176 ± 552	1241 ± 582	1032 ± 456.1	0.149	1439 ± 553	1018 ± 380.8	900.0 ± 522.1	≤0.001
erLF	14.7 ± 7.4	16.3 ± 7.7	10.5 ± 4.9	≤0.001	18.6 ± 7.7	11.9 ± 3.1	11.2 ± 7.1	≤0.001

Data are presented as mean (±standard deviation). NLF = normal liver function, LDF = liver dysfunction, PDR = ICR plasma disappearance rate, HBP = hepatobiliary phase, RR = reduction rate, LV = liver volume, eLF = estimated liver function, Rlv = remaining liver volume, erLF = estimated remaining liver function.

**Table 2:** Comparison of the proposed model to differentiate between the PNLF and PLDF groups to the ICG test.

Model	Sensitivity	Specificity	PPV	NPV	Accuracy	AUC
Liver function based on preoperative ICG [15]	0.53	0.77	0.46	0.82	0.70	0.73
Proposed model for estimating remaining postoperative liver function	0.53	0.89	0.63	0.84	0.79	0.75

PPV = positive predictive value, NPV = negative predictive value, AUC = area under the receiver curve.

## Data Availability

The data presented in this study are available on reasonable request from the corresponding author. Please note that there may be restrictions related to the pharmaceutical product featured in this study, and access to these specific data may require approval from Bayer AG.
